# Extra-Hepatic Manifestations of Hepatitis E Virus (HEV): A Narrative Review on Meningoencephalitis

**DOI:** 10.3390/life16081377

**Published:** 2026-08-20

**Authors:** Gaetano Scotto, Vincenzina Fazio, Mahmooda Kazmi, Sadia Khalil, Alessia Franza, Salvatore Massa

**Affiliations:** 1 Department of Biomedical Sciences, University of Foggia, 71121 Foggia, Italy; gaetano.scotto@unifg.it (G.S.); enzafazio@gmail.com (V.F.); 2 Department of Microbiology, Jinnah University for Women, Karachi 75300, Pakistan; mahmoodakzm2@gmail.com; 3 Department of Microbiology, Federal Urdu University of Arts, Science and Technology (FUUAST), Karachi 75300, Pakistan; sadia.khalil@fuuast.edu.pk; 4 Department of Agricultural, Food and Environmental Sciences, University of Foggia, 71121 Foggia, Italy; alessia_franza.542647@unifg.it

**Keywords:** hepatitis E virus, meningoencephalitis, neurological manifestations, neurotropism, blood–brain barrier, Guillain–Barré syndrome, ribavirin, genotype 3, cerebrospinal fluid, viral compartmentalization

## Abstract

Hepatitis E virus (HEV) infection, once regarded as a self-limiting hepatic disease confined to endemic regions, is now recognized as a systemic condition with a broad spectrum of extrahepatic manifestations. Among these, neurological complications—including peripheral neuropathies such as Guillain–Barré syndrome and neuralgic amyotrophy, and central nervous system involvement such as meningitis, encephalitis, and meningoencephalitis—are the most clinically relevant. Meningoencephalitis, though less common, is the most severe and underdiagnosed neurological expression of HEV infection. This narrative review examines current evidence on HEV-associated meningoencephalitis, covering epidemiology, pathogenesis, clinical features, and therapeutic strategies. Neurological disease is dominated by genotype 3 and frequently anicteric, with symptoms preceding or masking hepatic involvement. Pathogenesis is multifactorial, combining direct viral neurotropism—supported by HEV RNA detection in cerebrospinal fluid and replication in induced pluripotent stem cell (iPSC)-derived neurons and brain organoids—with immune-mediated mechanisms such as molecular mimicry and microglia-driven neuroinflammation. Diagnosis is often delayed by the absence of jaundice and low clinical suspicion, even though HEV remains an unrecognized cause in many unexplained encephalitis cases in high-income settings. Ribavirin remains the antiviral mainstay, but viral persistence within CNS sanctuary sites raises concerns about treatment adequacy and relapse; emerging antiviral and immunomodulatory strategies remain under investigation. HEV should be systematically considered in the diagnostic workup of unexplained neurological syndromes, regardless of overt hepatitis; heightened clinical awareness remains the most immediately actionable step toward reducing the burden of this still under-recognized condition.

## 1. Introduction

Hepatitis E virus (HEV) is one of the leading causes of acute viral hepatitis worldwide, accounting for millions of infections annually. Historically, HEV was regarded as a hepatotropic pathogen primarily associated with self-limiting acute hepatitis and large waterborne outbreaks in endemic regions [1,2]. However, growing clinical and experimental evidence has substantially expanded our understanding of HEV biology and disease spectrum. In addition to hepatic injury, HEV infection is now recognized as a systemic disease capable of affecting multiple extrahepatic organs and tissues [3].

Among the extrahepatic manifestations described to date, neurological complications represent the most frequently reported and clinically significant group [4]. These manifestations encompass a broad spectrum of disorders involving both the peripheral and central nervous systems, including neuralgic amyotrophy, Guillain–Barré syndrome, transverse myelitis, encephalitis, meningitis, and meningoencephalitis [4,5,6,7]. Importantly, neurological disease may occur in the absence of clinically apparent hepatitis, thereby contributing to underdiagnosis and delayed recognition of HEV infection [8]. Indeed, asymptomatic HEV infection is common, and viraemic blood donors frequently exhibit normal liver function tests despite active infection [9].

The increasing number of reports describing HEV-associated central nervous system (CNS) involvement has raised important questions regarding the neuroinvasive and neurotropic properties of the virus. Detection of HEV RNA in cerebrospinal fluid [10], evidence of viral compartmentalization between serum and CNS [11,12], and experimental studies demonstrating viral replication in neuronal cells [13] collectively support the concept that HEV can directly interact with neural tissues. Experimental evidence further suggests that HEV can cross the blood–brain barrier and establish compartmentalized infection within the CNS [14]. At the same time, immune-mediated mechanisms appear to contribute substantially to neurological injury, suggesting a multifactorial pathogenesis [15].

Among CNS manifestations, meningoencephalitis represents one of the most severe and clinically challenging presentations. Although relatively uncommon, HEV-associated meningoencephalitis has been reported in both immunocompetent and immunocompromised individuals and may result in significant morbidity [4]. The diagnosis remains difficult because clinical and radiological findings are often nonspecific and routine diagnostic algorithms do not usually include HEV testing [16,17].

This narrative review summarizes current knowledge regarding the epidemiology, pathogenesis, clinical presentation, diagnosis, and therapeutic management of HEV-associated neurological disease, with particular emphasis on meningoencephalitis. Special attention is given to recent advances supporting HEV neurotropism, CNS compartmentalization, and emerging therapeutic perspectives. Given that HEV is estimated to infect approximately 20 million people worldwide each year, a better understanding of its neurological consequences represents an important clinical and public health priority [18,19]. A list of the abbreviations used throughout this manuscript is provided at the end of the article, before the reference list.

## 2. Genome Organization and Structural Forms of Hepatitis E Virus

The HEV is a small positive-sense single-stranded RNA (+ssRNA) virus with an approximately 7.2 kb genome and an icosahedral capsid of 27–34 nm in diameter [20,21]. A distinctive biological feature of HEV is its ability to circulate in two structurally distinct forms depending on the biological compartment. In blood, virions are cloaked by a host-derived lipid membrane, forming quasi-enveloped particles, whereas in bile and feces they appear as non-enveloped virions [22].

**Figure 1:**
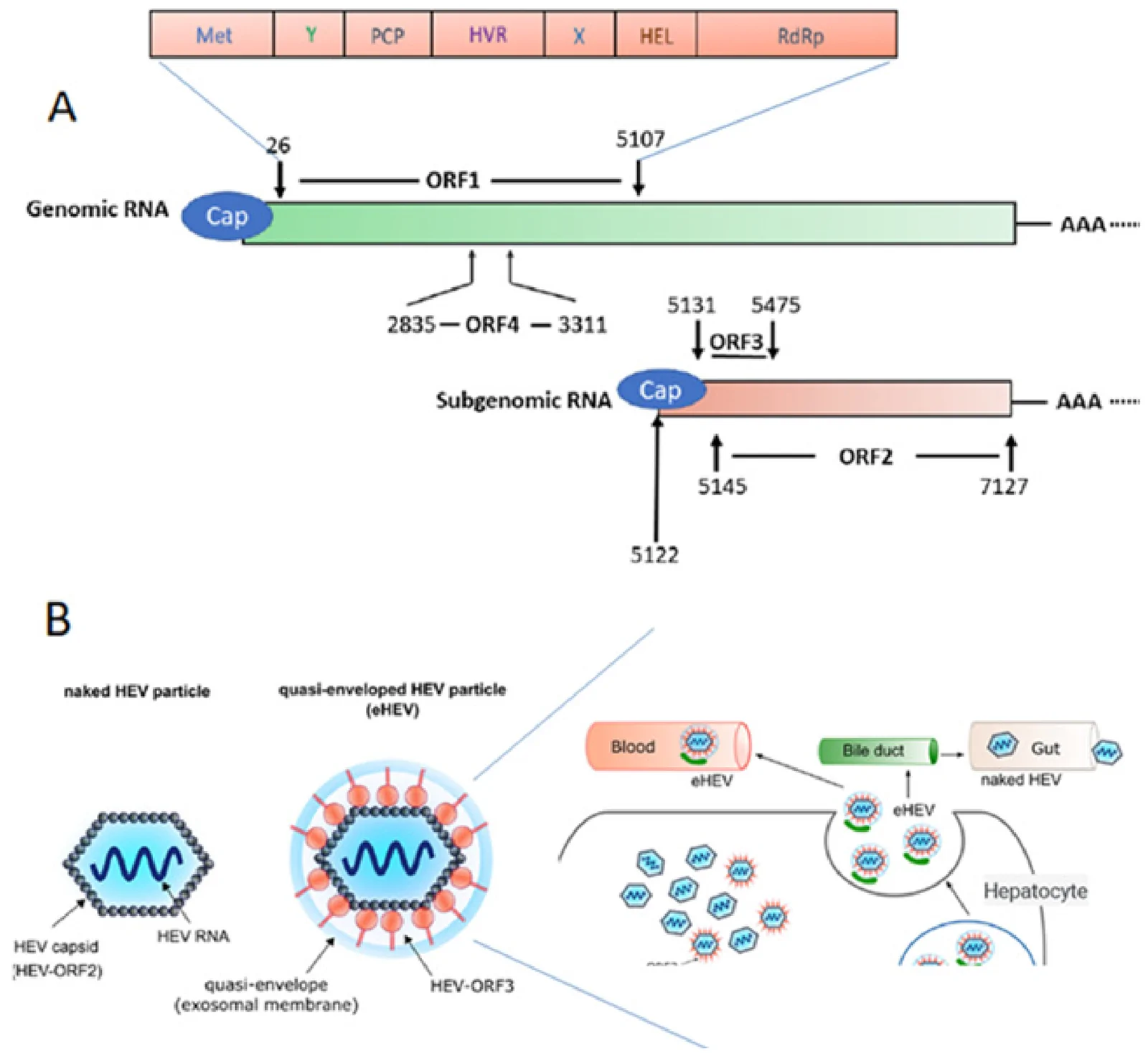
(**A**) Organization of the HEV genome (7.2 kb positive-sense RNA). The genome contains a 5′ non-coding region (NCR) capped with 7-methylguanosine (7 mG) and a 3′ NCR followed by a polyadenylated [poly(A)] tail, and includes three open reading frames (ORFs). ORF1 encodes the non-structural polyprotein; ORF2 encodes the capsid protein; and ORF3 encodes a small multifunctional phosphoprotein required for virion release through the ESCRT (Endosomal Sorting Complex Required for Transport) pathway. The numbers above and below the boxes indicate nucleotide positions. ORF1 spans nucleotides (nt) 26–5107 and contains several putative domains: MT, methyltransferase; Y domain; papain-like cysteine protease (PCP); HVR, hypervariable region; X domain; Hel, helicase; and RdRp, RNA-dependent RNA polymerase. In genotype 1 strains, an additional putative ORF4 has been identified within the ORF1 coding region. (**B**) Structural forms and release pathway of HEV particles. (**Left**) HEV exists as a non-enveloped (naked) virion, composed of the ORF2 capsid enclosing the viral RNA, or as a quasi-enveloped particle (eHEV) surrounded by a host-derived lipid membrane. (**Right**) During egress from infected hepatocytes, HEV acquires an exosome-derived membrane in an ORF3- and ESCRT-dependent process and is released into the bloodstream as eHEV. When these particles enter the biliary canaliculi and pass through the bile duct, bile salts remove the membrane, producing naked virions that reach the intestine and are excreted in feces. Adapted from [26].

The viral genome is capped at the 5′ end with a 7-methylguanosine cap and ends with a 3′ poly(A) tail, both enhancing RNA stability and translational efficiency [23]. It contains three partially overlapping open reading frames (ORF1, ORF2, ORF3), encoding non-structural proteins, the capsid protein, and a small multifunctional phosphoprotein, respectively [22]. These are flanked by short non-coding regions (NCRs) involved in replication and translation [21]. A fourth open reading frame (ORF4), present only in genotype 1 strains, is embedded within ORF1 and is expressed under stress conditions [24,25] (Figure 1A).

### 2.1. Genomic RNA, Regulatory Elements, and Subgenomic Transcription

ORF1 begins shortly downstream of the 5′ NCR and spans ~5081 nucleotides [26]. A junction region separates the replicase-coding ORF1 from the structural region containing ORF2 and ORF3. These two ORFs overlap and are translated from a bicistronic subgenomic RNA (sgRNA) of ~2.2 kb generated during replication [27].

The sgRNA drives high-level expression of ORF2 and ORF3, ensuring efficient virion assembly and release [27]. Replication is regulated by cis-reactive RNA elements located in the junction region and 3′ NCR, which recruit the RNA-dependent RNA polymerase (RdRp) and promote sgRNA synthesis. The 5′ NCR also contains sequences implicated in capsid interaction and virion assembly [21].

### 2.2. ORF1-Encoded Polyprotein

ORF1 encodes a large multifunctional polyprotein of ~1690 amino acids (~186–190 kDa) containing several domains: methyltransferase (Met), the Y domain (a conserved region of uncertain function that is thought to contribute to viral replication efficiency), papain-like cysteine protease (PCP), hypervariable region (HVR), the X (macro) domain, helicase (Hel), and RNA-dependent RNA polymerase (RdRp) [28,29].

The methyltransferase domain mediates 5′ RNA capping, while the Y domain enhances replication efficiency [30]. The PCP domain antagonizes innate immunity through deubiquitinase activity targeting RIG-I (Retinoic acid-Inducible Gene I) and TBK1 (TANK-binding kinase 1) [29]. The HVR increases replication and protein expression, and the X domain modulates interferon signaling by interfering with IRF-3 phosphorylation [31]. The helicase supports RNA unwinding and capping-related activities, while the RdRp catalyzes RNA synthesis using the 3′ genome end as a template [29].

Whether ORF1 is processed into individual proteins or functions as a single replicative complex remains unresolved [32].

### 2.3. ORF2: Capsid Protein and Virion Architecture

ORF2 encodes the capsid protein, the main structural component of the virion and the primary target of neutralizing antibodies. Multiple ORF2 forms are produced during infection, including an intracellular infectious form (ORF2i) and two secreted forms (glycosylated ORF2g and cleaved ORF2c) not incorporated into virions [33].

The secreted forms are highly abundant in serum and likely function as antigenic decoys, binding anti-capsid antibodies and reducing neutralization of infectious particles [33].

### 2.4. ORF3: Multifunctional Protein Involved in Virion Release and Quasi-Envelopment

ORF3 encodes a small ~13 kDa phosphoprotein essential for late stages of the viral life cycle. It interacts with host vesicular trafficking pathways and recruits ESCRT components, including TSG101 (Tumor Susceptibility Gene 101), promoting virion egress from hepatocytes [34].

This process enables acquisition of a host-derived lipid envelope, resulting in quasi-enveloped particles (eHEV). ORF3 also functions as a viroporin, forming ion channels that facilitate viral release [35].

### 2.5. ORF4: An Additional Replication Factor in Genotype 1

ORF4 is present only in genotype 1 HEV and is translated via an internal ribosome entry site (IRES)-like mechanism under endoplasmic reticulum stress conditions [26]. The ORF4 protein interacts with viral replication machinery, including RdRp and helicase, enhancing RNA synthesis efficiency [36].

### 2.6. Structural Forms of HEV Particles and Immune Evasion

HEV exists in two biologically distinct particle forms. In blood, virions are quasi-enveloped (eHEV) (Figure 1B), cloaked by a host-derived lipid membrane lacking viral glycoproteins [35]. This membrane masks the ORF2 capsid, limiting recognition by neutralizing antibodies and facilitating immune evasion during systemic dissemination [37].

In bile and feces, the lipid envelope is removed by bile salts, generating non-enveloped virions with fully exposed capsids These particles exhibit higher infectivity and are optimized for fecal–oral transmission between hosts [37]. This dual-particle strategy enables immune evasion within the host and efficient transmission between hosts.

## 3. Taxonomy

The taxonomy of HEV has evolved considerably since its first recognition during an epidemic of non-A, non-B hepatitis in the Kashmir Valley in 1978 [38], reflecting advances in molecular and phylogenetic characterization.

**Figure 2:**
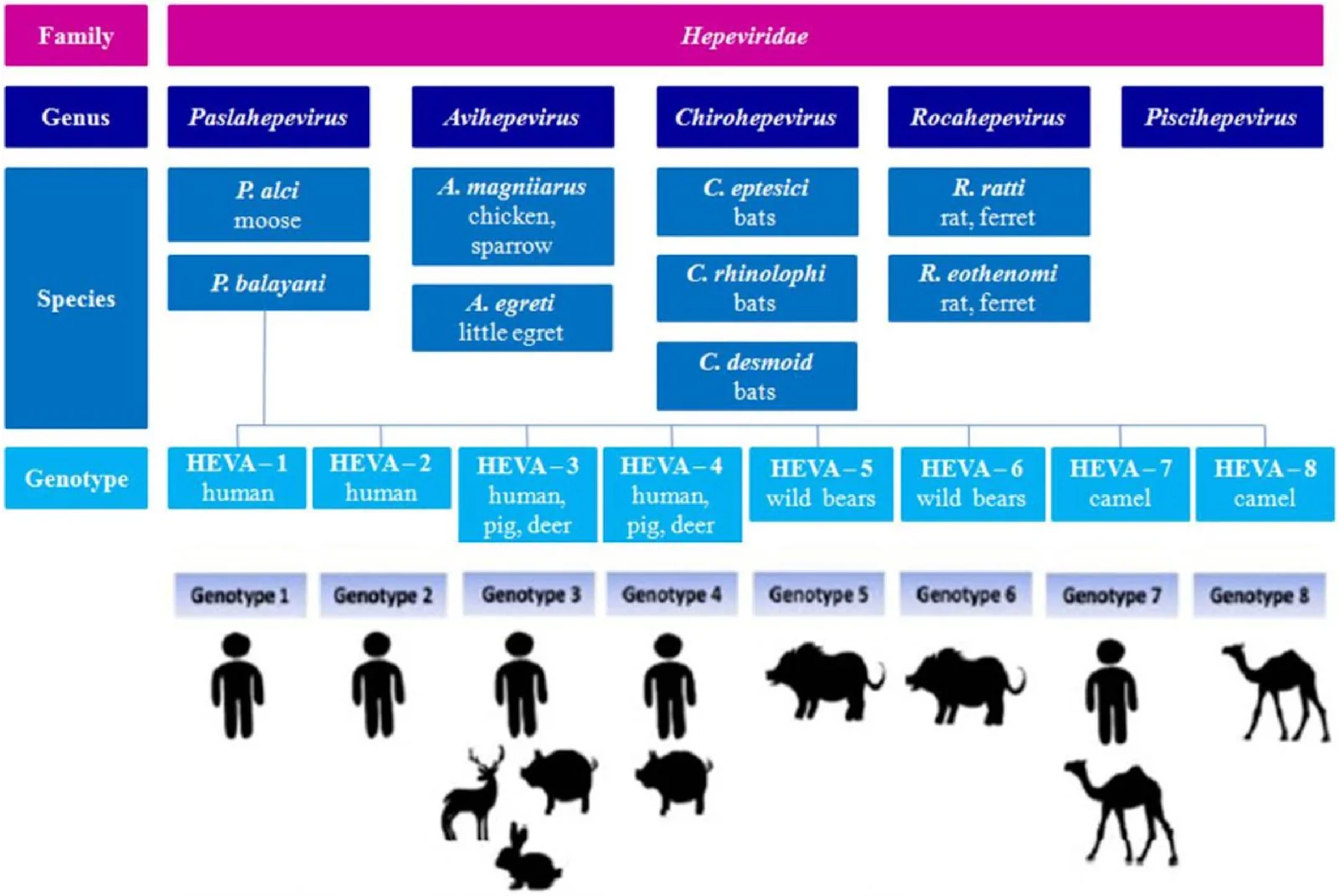
Taxonomic classification of hepatitis E virus (HEV) within the family Hepeviridae. The family *Hepeviridae* currently comprises two subfamilies (*Orthohepevirinae* and *Parahepevirinae*), five genera (*Paslahepevirus*, *Avihepevirus*, *Chirohepevirus*, *Rocahepevirus*, and *Piscihepevirus*), and ten recognized species [39]. For clarity, the two subfamilies are not shown in the figure. The genus *Paslahepevirus* includes two species, *Paslahepevirus balayani* and *Paslahepevirus alci*. The species *P. balayani* is divided into eight genotypes (HEV-A1–HEV-A8); the prefix “A” refers to the former species designation *Orthohepevirus A* used in earlier HEV taxonomy. Among these, genotypes HEV-A1 to HEV-A4 and HEV-A7 have been reported to infect humans. HEV-A1 and HEV-A2 infect humans exclusively, whereas HEV-A3 and HEV-A4 infect humans and several animal species (including pigs, wild boar, and deer). Genotypes HEV-A5 and HEV-A6 have been detected mainly in wild boar, while HEV-A7 (with a single human infection up to now) and HEV-A8 have been identified in camels. The genus *Piscihepevirus* comprises a single species (*Piscihepevirus heenan*), which infects fish and is not represented in the figure. Adapted from [22].

According to the International Committee on Taxonomy of Viruses [39], HEV is classified within the family Hepeviridae, which comprises two subfamilies: Orthohepevirinae and Parahepevirinae [40]. Orthohepevirinae includes genera infecting mammals and birds (*Avihepevirus*, *Chirohepevirus*, *Paslahepevirus*, and *Rocahepevirus*), while Parahepevirinae includes fish-associated viruses classified within *Piscihepevirus* [23]. This taxonomic organization is summarized in Figure 2.

Human-pathogenic HEV strains belong primarily to the genus *Paslahepevirus*, particularly the species *Paslahepevirus balayani*, which includes eight genotypes (HEV-A1 to HEV-A8) [23]. Genotypes HEV-1 and HEV-2 are strictly human-associated and responsible for large waterborne outbreaks in regions with poor sanitation infrastructure [41,42,43]. In contrast, HEV-3 and HEV-4 are zoonotic, infecting humans and multiple animal reservoirs, including swine, wild boar, deer, rabbits, and others, and are responsible for sporadic infections worldwide [43].

Additional genotypes within *P. balayani* have been identified in animal hosts: HEV-5 and HEV-6 in wild boar, HEV-7 in dromedary camels [44,45] and HEV-8 in Bactrian camels [46]. Notably, HEV-7 has been associated with zoonotic transmission to humans through camel-derived products [47].

Beyond *Paslahepevirus*, human infections have also been reported with *Rocahepevirus ratti* (HEV-C1), primarily maintained in rodents but capable of crossing species barriers [48]. Within this lineage, HEV-C1 and HEV-C2 have been described in rodents and carnivores such as ferrets and minks, respectively, highlighting the zoonotic potential of the virus [23].

Overall, HEV taxonomy reflects a complex evolutionary landscape characterized by multiple host-associated lineages and ongoing zoonotic emergence across diverse animal reservoirs [23].

## 4. Global Epidemiology

### 4.1. HEV Genotype Distribution Worldwide

The World Health Organization (WHO) estimates that there were approximately 19.47 million cases of acute hepatitis E globally in 2021, resulting in 3450 deaths (World Health Organization. Hepatitis E [Internet]. Available from: https://www.who.int/news-room/fact-sheets/detail/hepatitis-e accessed on 1 June 2026). The largest outbreak of HEV infection occurred in Namibia from late 2017 to early 2020 [49]. Smaller HEV outbreaks in Burkina Faso occurred in 2020 and in South Sudan from mid-2021 to early 2022 following heavy rainfall and flooding in that area [50,51].

Serological and molecular studies have demonstrated that HEV is widely distributed worldwide and represents the leading cause of enterically transmitted viral hepatitis.

**Figure 3:**
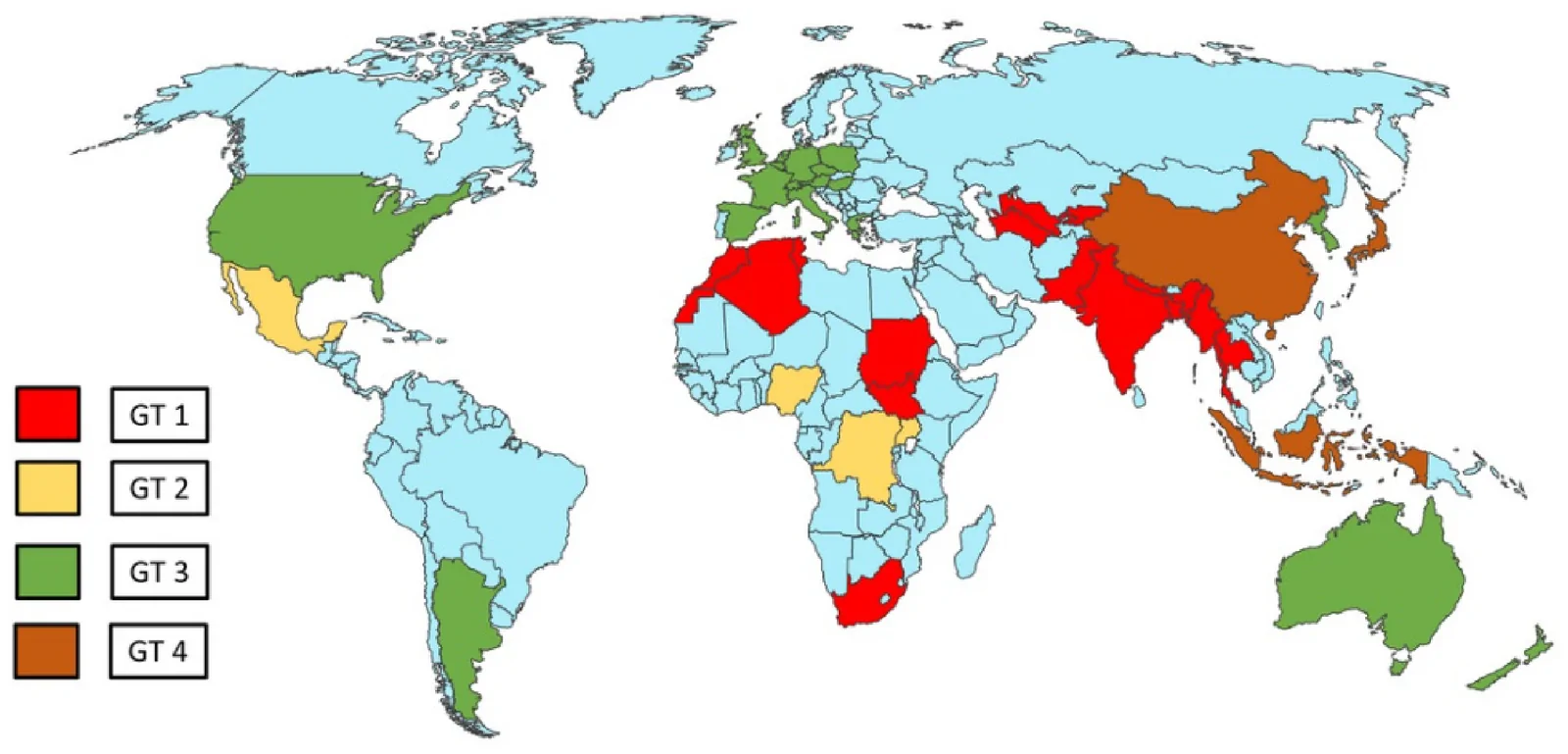
Global distribution of predominant HEV genotypes. HEV-1 and HEV-2 (red and yellow) are mainly found in low- and middle-income regions of Asia and Africa, where they are responsible for large waterborne outbreaks and sporadic cases linked to poor sanitation. HEV-3 (green) predominates in Europe, North America, and parts of South America, while HEV-4 (brown) is mainly distributed in East and Southeast Asia, particularly in China and neighboring countries. In several regions, co-circulation of multiple genotypes has been documented, reflecting differences in transmission routes, reservoirs, and socioeconomic conditions. Notably, the apparent absence of documented HEV-3 activity in the Amazon basin, despite the region’s extensive limitations in sanitation infrastructure, likely reflects a paucity of systematic surveillance and molecular genotyping data rather than a true absence of circulation, underscoring a gap in epidemiological coverage for this area. Adapted from [53].

The epidemiology of HEV is strongly influenced by genotype distribution, which varies geographically (Figure 3) and correlates with distinct transmission routes, reservoirs, and clinical outcomes [52].

In general, genotypes 1 and 2 (G1, G2) are predominant in low- and middle-income regions, particularly in Asia and Africa, where they are transmitted via the fecal–oral route through contaminated water and are responsible for both large outbreaks and sporadic cases [53]. These genotypes are associated with a higher disease burden and more severe clinical outcomes, especially among pregnant women, in whom mortality rates may reach up to 25% [54,55]. G1 is the dominant genotype across South Asia, including India, Nepal, Bangladesh, and Pakistan, where it accounts for the vast majority of both epidemic and sporadic hepatitis E cases, showing a high degree of genetic homogeneity [56]. In Africa, G1 is also widely distributed, although G2 has been identified in several West and Central African countries, sometimes co-circulating with G1 and contributing to regional variability in genotype distribution [43,57].

In contrast, genotypes 3 and 4 (G3, G4) are mainly observed in high-income and industrialized regions, including Europe, North America, and parts of East and Southeast Asia, such as Japan [58], Korea [59], and Singapore [60].

These genotypes are zoonotic, with domestic pigs and wild boars serving as the primary reservoirs, and transmission is typically associated with the consumption of raw or undercooked meat or direct contact with infected animals [61,62]. G3 is the predominant genotype in Europe and North America and represents the main cause of autochthonous infections in these areas, including cases identified in individuals without travel history [57]. It is also associated with chronic hepatitis in immunocompromised patients and with a range of extrahepatic manifestations, particularly neurological disorders [63]. Although G4 is more frequently reported in East Asia, especially in China, Japan, and Southeast Asian countries, increasing evidence indicates its sporadic emergence in Europe, suggesting possible introduction through food importation or travel-related exposure [64]. Although HEV-3 and HEV-4 co-circulate in parts of Europe, HEV-3 remains the predominant genotype.

Overall, the global distribution of HEV genotypes reflects a dual epidemiological pattern: waterborne transmission driven by G1 and G2 in resource-limited settings, and zoonotic, foodborne transmission driven mainly by G3 and, to a lesser extent, G4 in industrialized countries [65].

Age-related epidemiological patterns have also received growing attention. Comprehensive overviews of global HEV epidemiology confirm that industrialized countries generally show lower anti-HEV IgG seroprevalence (1–52%) than developing regions (4–94%), with seroprevalence increasing progressively with age in most populations [56]. Using Global Burden of Disease (GBD) 2021 data, two recent studies have specifically examined the burden of acute hepatitis E among older adults, a population previously understudied despite its heightened vulnerability to severe outcomes. Wu et al. [66] reported that, among individuals aged 60 years and older, the global age-standardized incidence of acute hepatitis E remained essentially stable between 1990 and 2021, while mortality declined significantly (average annual percentage change, AAPC: −2.9%); incidence increased substantially within the oldest age groups (75–94 years) and was consistently higher in females than males across all age groups, with the greatest disparity observed in low socio-demographic index (SDI) countries. Consistently, Ma et al. [67], analyzing adults aged 65 years and older, found that the global age-standardized incidence rate increased by approximately 1.5% annually over the same period, whereas mortality and disability-adjusted life years (DALYs) declined significantly; the 95-years-and-older age group bore the highest overall disease burden, and projections through 2050 suggest that incidence in this population will continue to rise even as mortality continues to fall. Both studies attribute the rising burden among the elderly, at least in part, to immunosenescence and an increasing prevalence of chronic comorbidities, factors that plausibly also contribute to the heightened susceptibility of older patients with HEV infection to extrahepatic and neurological complications.

### 4.2. Epidemiology of HEV-Associated Neurological Manifestations

#### 4.2.1. Peripheral Nervous System Manifestations

The peripheral nervous system represents the most frequently reported extrahepatic neurological target of HEV infection. Neurological manifestations have been estimated to occur in approximately 5–16% of patients with acute HEV infection, although their reported frequency varies according to the study population, geographical setting, genotype distribution, and diagnostic approach [16,68].

Most neurological cases reported in Europe and other high-income countries are associated with genotype 3 (HEV-3), which is the predominant circulating strain in these regions [2,69]. Conversely, neurological complications related to genotypes 1 and 4 have been described less frequently in endemic areas, although this difference may partly reflect limited diagnostic availability and underrecognition rather than true epidemiological variation [70].

Neuralgic amyotrophy (NA), also known as Parsonage–Turner syndrome, is one of the most characteristic peripheral neurological complications associated with HEV infection. This disorder mainly affects the brachial plexus and cervical nerve roots and is characterized by sudden-onset severe shoulder or arm pain, followed by asymmetric muscle weakness and amyotrophy [71]. A marked male predominance has been consistently reported, with men representing up to 87% of affected patients [72]. Although the mechanisms underlying this sex difference remain unclear, hormonal, immunological, and occupational factors have been proposed. HEV-associated NA is predominantly linked to HEV genotype 3 and frequently occurs without clinically evident hepatitis, which may delay diagnosis.

Guillain–Barré syndrome (GBS) is another well-recognized peripheral neurological manifestation of HEV infection. GBS is an acute inflammatory polyradiculoneuropathy characterized by rapidly progressive bilateral weakness, sensory disturbances, and reduced or absent tendon reflexes. Cerebrospinal fluid typically shows cytoalbuminologic dissociation. HEV-associated GBS is thought to result mainly from immune-mediated mechanisms, including molecular mimicry and the production of anti-ganglioside antibodies such as GM1 and GM2, similarly to other infection-related forms of GBS [73,74,75,76]. However, the precise mechanisms involved remain incompletely understood.

Several epidemiological studies support an association between HEV infection and GBS. Acute HEV infection has been identified in approximately 5% of patients with GBS in the Netherlands, 4.8% in Japan, and up to 11% in Bangladesh, with higher frequencies than those observed in control populations [77,78,79]. A review of reported cases demonstrated a male predominance, a mean age of approximately 51 years, and HEV-3 as the predominant genotype in European cases [80,81,82,83]. Clinically, HEV-associated GBS does not appear to differ substantially from GBS caused by other infectious or immune triggers.

Peripheral neuropathies other than NA and GBS are less frequently reported but likely underrecognized. Documented phenotypes include sensory neuropathies, sensorimotor neuropathies, mononeuropathies, and mononeuritis multiplex [82,83]. In a French series of immunocompetent patients with acute autochthonous HEV infection, mononeuritis multiplex was characterized by asymmetric neuropathic pain, paresthesia, and hypo- or areflexia, often in the absence of jaundice despite abnormal liver enzymes [82]. Most patients showed favorable neurological outcomes after clearance of HEV infection, although persistent deficits were occasionally observed.

Additional evidence comes from a large prospective Chinese case–control study including patients with acute non-traumatic neurological disorders, in which acute HEV infection was detected in a small proportion of subjects, including one patient with peripheral neuropathy without clinically apparent hepatitis [83]. Although the available epidemiological evidence remains limited, these observations support a possible association between HEV infection and a broader spectrum of peripheral nerve disorders.

Other autoimmune neurological conditions have also been described in association with HEV infection, although evidence remains preliminary. A pilot study by Wang et al. [84] investigated the prevalence of current or recent HEV infection in 188 patients with newly diagnosed myasthenia gravis (MG). Anti-HEV IgM positivity was detected in 5.3% of patients, while HEV RNA was identified in a subset of cases, suggesting that HEV infection may represent a potential trigger or contributing factor in susceptible individuals. However, the study did not demonstrate clear differences in disease severity or outcome compared with HEV-negative patients, and a causal relationship remains unproven.

Similarly, chronic inflammatory demyelinating polyneuropathy (CIDP) has been reported in association with HEV, although the available data are scarce. A recent retrospective study [85] evaluating neurological patients with previously unexplained disorders found that evidence of HEV exposure was relatively frequent among patients with inflammatory demyelinating neuropathies, whereas acute HEV infection was uncommon. These findings suggest that HEV may occasionally act as a trigger of immune-mediated peripheral neuropathies, but further studies are required to clarify its clinical relevance.

Overall, current evidence indicates that HEV-associated peripheral nervous system involvement extends beyond the classical manifestations of neuralgic amyotrophy and Guillain–Barré syndrome, including a heterogeneous group of neuropathic and autoimmune disorders. Although the strength of evidence varies among different conditions, HEV should be considered in the diagnostic evaluation of patients with unexplained acute or subacute peripheral neurological syndromes, particularly when accompanied by abnormal liver enzymes or epidemiological risk factors.

#### 4.2.2. Central Nervous System Involvement

Central nervous system (CNS) infections associated with hepatitis E virus (HEV)—including meningitis, encephalitis, meningoencephalitis, and, more rarely, myelitis—are increasingly recognized but remain considerably less common than peripheral nervous system manifestations [72]. Available data suggest that CNS involvement accounts for approximately 1–5% of neurological complications observed during HEV infection, although reliable epidemiological estimates remain difficult because current evidence is largely derived from case reports and small observational series [16,69]. Most reported cases originate from Europe and are associated with genotype 3 (HEV-3), the predominant genotype in industrialized countries, although sporadic CNS manifestations linked to genotypes 1 and 4 have also been described [72].

Among CNS manifestations, encephalitis appears to be the most frequently reported clinical syndrome, followed by meningitis and meningoencephalitis [63]. In the review by Willauer et al. [70] including published neurological cases of HEV infection, meningoencephalitis was identified in six patients with a mean age of 44 years and no evident sex predominance; notably, all reported patients were anicteric despite abnormal liver enzyme levels, emphasizing that the absence of clinically overt hepatitis does not exclude HEV-associated CNS disease [83,86,87]. In most cases, HEV RNA was detectable in serum, whereas detection in cerebrospinal fluid (CSF) was inconsistent, being identified in only a subset of tested patients [72]. HEV meningitis usually presents with nonspecific symptoms such as fever, headache, photophobia, and neck stiffness, accompanied by lymphocytic pleocytosis and mild protein elevation in CSF. Clinical presentation is therefore largely indistinguishable from other forms of viral aseptic meningitis. In selected cases, HEV RNA has been detected directly in CSF, providing evidence supporting viral neuroinvasion and local CNS replication [11]. However, negative CSF PCR does not exclude the diagnosis, as viral RNA detection in CSF appears variable and probably depends on timing of lumbar puncture, viral load, and compartmentalization of infection [16]. Consistent with this variability, a large multicenter Vietnamese cohort study found no HEV RNA in 330 CSF samples from patients with suspected CNS infections, suggesting a limited role for HEV in this population and underscoring substantial geographic heterogeneity in reported HEV neuroinvasion [88].

HEV encephalitis is characterized by altered mental status, confusion, cognitive impairment, seizures, focal neurological deficits, or reduced consciousness [4,72]. Neuroimaging findings are heterogeneous and nonspecific. Brain MRI (Magnetic Resonance Imaging) may demonstrate T2/FLAIR (Fluid Attenuated Inversion Recovery) hyperintense lesions involving cortical, subcortical, temporal, frontal, thalamic, or cerebellar regions, although imaging can remain normal in a substantial proportion of patients [63]. This radiological heterogeneity likely reflects the multifactorial pathogenesis of HEV-related CNS injury, in which direct viral neurotropism and immune-mediated inflammatory mechanisms may coexist or occur sequentially. Increasing evidence supports the neurotropic potential of HEV, including reports demonstrating viral compartmentalization between serum and CSF and the direct identification of HEV RNA within the CNS [4,12,89].

Meningoencephalitis represents the most severe and clinically relevant CNS manifestation of HEV infection and constitutes the primary focus of this review. Reported cases describe highly variable clinical presentations, ranging from mild confusion and fever to severe encephalopathy with seizures and persistent neurological sequelae [2,83,86,90,91]. Although outcomes are generally favorable, incomplete neurological recovery and fatal cases have occasionally been reported. Most published patients were infected with HEV-3 and lacked clinically apparent jaundice, further supporting the concept that HEV-associated CNS disease may occur independently of severe hepatic involvement [72]. Recent advances in molecular diagnostics, including metagenomic next-generation sequencing (mNGS), have further strengthened the causal association between HEV and CNS infection by enabling direct viral identification in CSF from otherwise unexplained meningoencephalitis cases [90].

Acute transverse myelitis (ATM) is another uncommon but increasingly recognized CNS complication of HEV infection. Patients typically present with acute motor weakness, sensory deficits, sphincter dysfunction, and a defined spinal sensory level [72,92]. Although evidence remains limited to isolated reports and small case series, detection of HEV RNA in both serum and CSF in several patients strongly supports a causal relationship [93,94]. More recently, chronic HEV infection associated with recurrent myelopathy and spinal cord atrophy has been described in immunocompromised hosts, suggesting that persistent CNS viral replication may occur in selected patients [95].

CNS involvement has been documented in both immunocompetent and immunocompromised individuals. While chronic infection and severe neurological disease appear more frequent in transplant recipients and other immunosuppressed patients, a substantial number of CNS cases have been reported in previously healthy subjects [2,16]. Moreover, unusual zoonotic HEV variants may also exhibit neuroinvasive potential. Notably, rat HEV-associated meningoencephalitis with detectable HEV RNA in CSF has been described in immunocompromised patients, further expanding the spectrum of HEV neurotropism [96]. In a South African cohort of people living with HIV, extrahepatic HEV RNA was similarly detected in CSF, providing additional evidence that HIV-related immunosuppression may facilitate detectable HEV replication within the CNS compartment [97], consistent with earlier reports of persistent HEV carriage in HIV-infected individuals more broadly [98].

The diagnosis of HEV-associated CNS disease remains challenging and is likely substantially underestimated. HEV testing is not routinely included in the diagnostic evaluation of patients presenting with meningitis or encephalitis of unknown origin, particularly in the absence of jaundice or clinically evident hepatitis. This issue is clinically relevant because a large proportion of encephalitis cases in Europe remain etiologically unexplained despite extensive diagnostic investigation [17]. Supporting this view, a prospective screening study of patients presenting with acute neurological disorders found a non-negligible proportion with evidence of HEV infection, reinforcing the case for systematic testing in this setting [99]. Current evidence therefore suggests that HEV serology and nucleic-acid testing in serum—and, when feasible, in CSF—should be systematically considered in patients with unexplained CNS inflammation, especially in the presence of elevated liver enzymes, immunosuppression, zoonotic exposure, or relevant epidemiological risk factors. Importantly, diagnosis should not rely exclusively on CSF PCR (polymerase chain reaction) because viral RNA is inconsistently detectable within the CNS; rather, diagnostic assessment should integrate serological findings, plasma or stool HEV RNA testing, neuroimaging, and clinicoradiological correlation [10,11,89]. Table 1 provides a summary overview of the main HEV-associated neurological manifestations discussed above, together with their principal clinical features, proposed pathogenetic mechanisms, and representative references.

## 5. Pathogenesis of HEV-Associated Neurological Injury

The mechanisms underlying HEV-associated neurological injury remain incompletely understood and are likely multifactorial. Current evidence supports the coexistence of two complementary pathogenic pathways: (i) immune-mediated neural injury and (ii) direct viral neurotropism with central nervous system (CNS) invasion and compartmentalized replication. Experimental studies, animal models, and clinical observations collectively indicate that HEV can disseminate beyond the liver, cross the blood–brain barrier (BBB), infect neural cells, and induce sustained neuroinflammatory responses. At the same time, several peripheral neurological syndromes appear to result predominantly from dysregulated host immune responses rather than direct viral replication within neural tissue [74,75,76,110,112].

**Figure 4:**
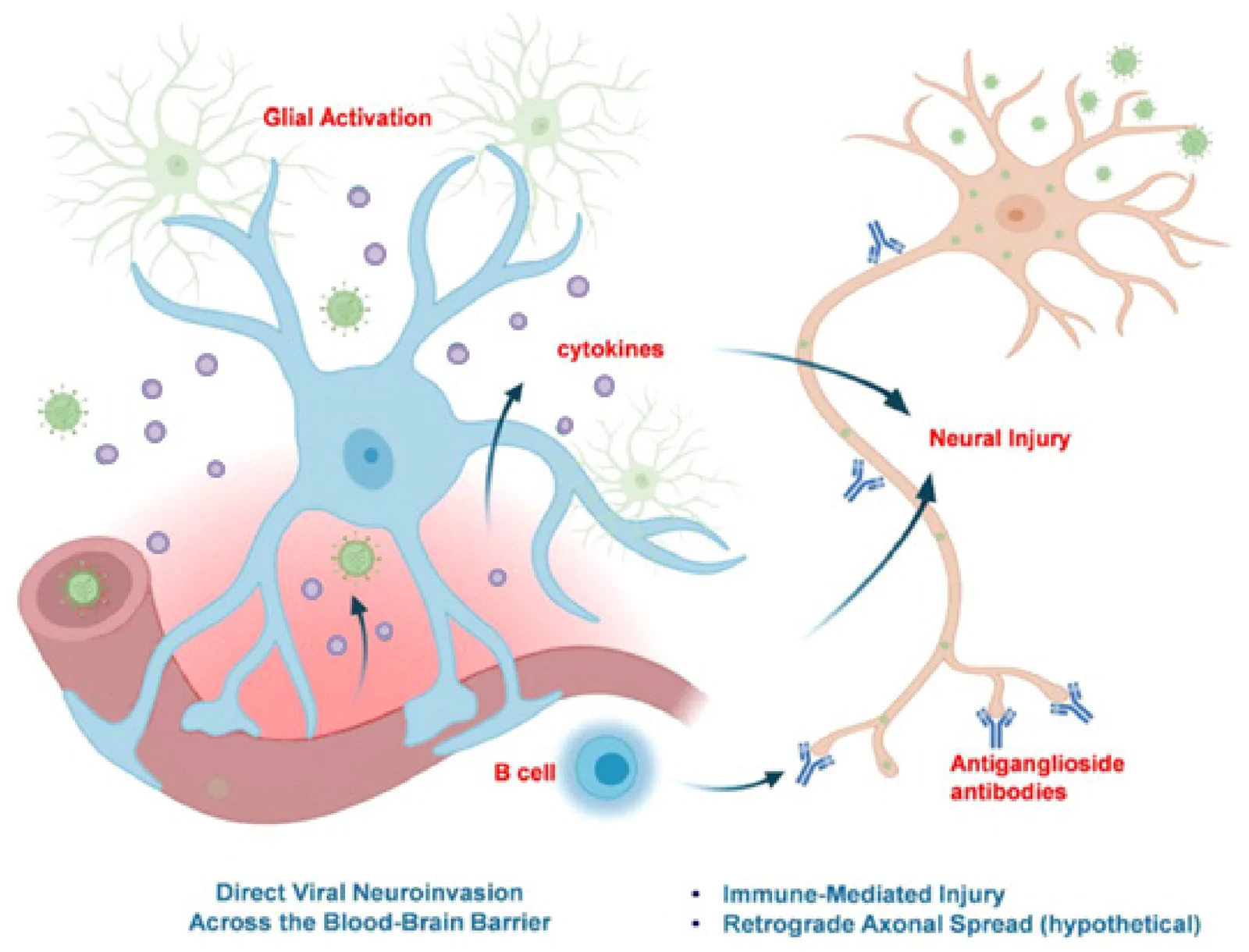
Proposed mechanisms of HEV-associated neuropathogenesis. The diagram illustrates the two complementary pathways of HEV-associated neuropathogenesis described in the text. Direct viral neuroinvasion (**left**) involves hematogenous entry across the blood–brain barrier (BBB), infection of neurovascular and glial cells, microglial activation, and release of pro-inflammatory cytokines (TNF-α, IL-1β, IFN-γ), leading to neuroinflammation and neuronal injury. Immune-mediated injury (**right**) predominantly affects the peripheral nervous system and is driven by dysregulated host immune responses, including B-cell activation, molecular mimicry, and production of anti-ganglioside antibodies (anti-GM1, anti-GM2) targeting peripheral nerve structures. A hypothetical retrograde axonal spread is depicted as a possible but unproven additional route of CNS entry. Together, these mechanisms highlight the multifactorial and heterogeneous nature of HEV-induced neurological disease. Reproduced with color modifications from [82].

Importantly, neurological manifestations may occur despite mild hepatic involvement or even after apparent systemic viral clearance, suggesting that neural injury may persist independently from liver disease through ongoing intrathecal immune activation and/or localized CNS viral persistence. Figure 4 summarizes the principal mechanisms proposed to contribute to HEV-associated neuropathogenesis.

### 5.1. Immune-Mediated Mechanisms

Increasing evidence supports a major role for immune-mediated injury in HEV-associated neurological disease, particularly in peripheral nervous system syndromes such as Guillain–Barré syndrome (GBS) and neuralgic amyotrophy (NA). One of the strongest arguments supporting this hypothesis is the significantly higher prevalence of neurological manifestations among immunocompetent patients compared with immunocompromised individuals (22.6% vs. 3.2%, *p* < 0.001) [112]. Moreover, patients developing NA or GBS following HEV infection are typically immunocompetent [110,112], suggesting that exaggerated or misdirected host immune responses substantially contribute to tissue damage.

The proposed mechanism resembles post-infectious autoimmune neuropathies mediated by molecular mimicry. Viral antigens may share structural similarities with gangliosides and other neural membrane components, leading to the generation of cross-reactive antibodies directed against peripheral nerve structures. Anti-ganglioside antibodies, particularly anti-GM1 and anti-GM2, have been detected in HEV patients with GBS and related neuropathies [74,75,76]. Similar observations were subsequently confirmed in additional studies, further supporting the role of humoral autoimmunity in HEV-associated neural injury [75,83].

Cell-mediated immune responses likely contribute as well. Following HEV infection, antigen-presenting cells activate CD4+ and CD8+ T-cell responses associated with production of pro-inflammatory cytokines including TNF-α, IL-1β, IL-6, IL-8, IL-12, and IFN-γ. These mediators may promote endothelial dysfunction, BBB disruption, and neural tissue injury. In particular, IFN-γ released by Th1 lymphocytes enhances macrophage and microglial activation, amplifying neuroinflammatory responses against myelin and axonal structures [85,113]. IL-17 produced by Th17 cells may further facilitate disruption of the blood–spinal cord barrier and recruitment of neutrophils into neural tissue, contributing to oxidative stress and oligodendrocyte damage [114].

Innate immune activation also appears to play an important role. Activated microglia recognize viral pathogen-associated molecular patterns through Toll-like receptors, leading to release of TNF-α, IL-1β, reactive oxygen species, nitric oxide, and other neurotoxic mediators [89,115]. Reactive astrogliosis may initially limit tissue injury but can subsequently impair glutamate homeostasis and promote excitotoxic neuronal damage [89,115].

In addition to cellular immune mechanisms, immune-complex-mediated injury may contribute to extrahepatic disease. Recent studies demonstrated that excess circulating HEV ORF2 capsid protein can form antigen–antibody immune complexes capable of inducing tissue inflammation even in the absence of productive local viral replication [89]. Similar mechanisms may plausibly contribute to some neurological manifestations associated with HEV infection.

### 5.2. Direct Viral Neurotropism and CNS Compartmentalization

Although immune-mediated mechanisms likely predominate in many peripheral neuropathies, increasing evidence indicates that HEV can directly infect the nervous system in at least a subset of patients. This concept is supported by convergent clinical, virological, and experimental observations demonstrating BBB disruption, CNS invasion, intrathecal viral persistence, and compartmentalized viral evolution.

Crossing of the BBB appears to represent the critical initial step in HEV neuroinvasion. Experimental studies demonstrated that HEV infection alters the expression of key tight-junction proteins, including claudin-5, occludin, and zonula occludens-1 (ZO-1), thereby increasing BBB permeability [8]. Pro-inflammatory cytokines such as TNF-α and IL-1β may further destabilize endothelial integrity and facilitate viral access to the CNS [116]. In vitro BBB models additionally showed that both quasi-enveloped and non-enveloped HEV particles can traverse endothelial barriers, supporting hematogenous dissemination as the principal route of neuroinvasion [82,117]. A “Trojan horse” mechanism involving infected monocytes or macrophages has also been proposed, although direct evidence for this pathway in HEV infection remains limited [116].

One of the strongest arguments supporting direct HEV neurotropism derives from the demonstration of genetically distinct viral populations within the cerebrospinal fluid (CSF) and serum of the same patient [118]. In a chronically infected kidney transplant recipient presenting with pyramidal syndrome, analysis of HEV quasispecies revealed significant divergence between serum-derived and CSF-derived viral strains, strongly suggesting compartmentalized CNS replication [118]. Subsequent reports confirmed persistent HEV RNA in CSF despite viral clearance from plasma and feces following ribavirin therapy. In these patients, intrathecal synthesis of anti-HEV IgG and mutations within the polyproline region suggested autonomous viral evolution within the CNS [13].

More recent next-generation sequencing studies further strengthened the concept of viral compartmentalization by demonstrating that HEV variants isolated from CSF may differ substantially from circulating blood strains, particularly within ORF1 and hypervariable regions [11]. These findings suggest that the CNS may function as an autonomous viral reservoir in which HEV undergoes local adaptation under distinct selective pressures. Such compartmentalization may facilitate viral persistence by enabling evasion from systemic immune surveillance and limiting antiviral efficacy within the CNS.

The concept of the CNS as a virological sanctuary has important clinical implications. HEV RNA may remain detectable in CSF long after apparent systemic recovery, indicating that neurological disease may persist independently from hepatic infection [13]. Inadequate penetration of antiviral agents across the BBB could therefore permit ongoing intrathecal replication despite clearance of systemic infection. This mechanism may help explain chronic or relapsing neurological manifestations observed in some patients with HEV-associated meningoencephalitis.

Direct cytopathic effects may additionally contribute to neural injury. Experimental studies demonstrated that HEV replication in neuronal cells induces neurite shortening, mitochondrial dysfunction, oxidative stress, and neuronal degeneration [15]. Human induced neurons infected with HEV exhibit relatively weak intrinsic antiviral responses, suggesting that neural tissue may constitute a permissive environment for persistent infection and sustained neuroinflammation.

### 5.3. Experimental Evidence Supporting HEV Neurotropism

**Figure 5:**
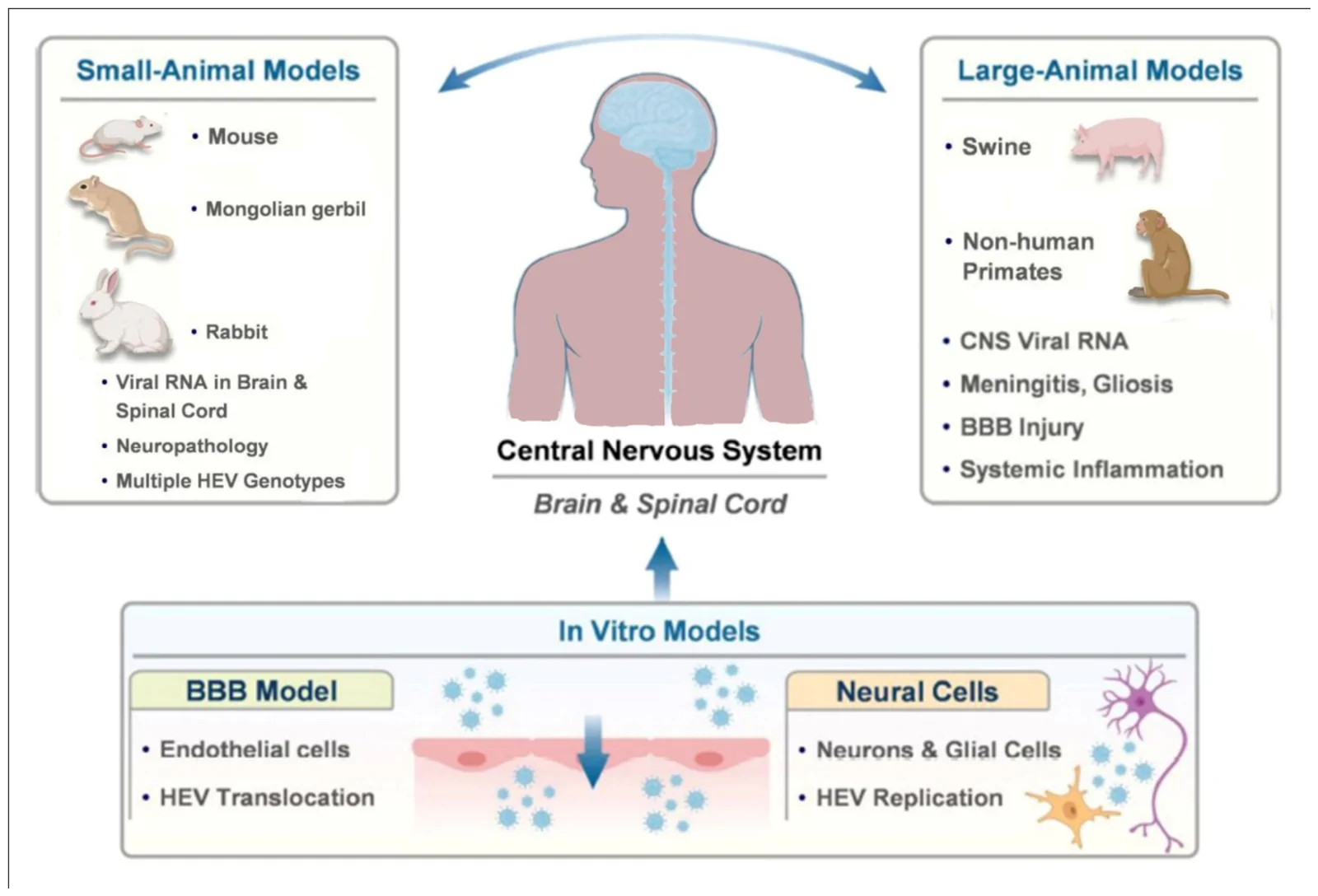
**Overview of experimental models supporting HEV neurotropism.** The diagram summarizes the principal in vivo and in vitro systems used to investigate HEV-induced neurological injury. Small-animal models (mouse, Mongolian gerbil, rabbit) have demonstrated viral RNA and ORF2 protein expression in brain and spinal cord tissues, along with neuropathological changes across multiple HEV genotypes. Large-animal models (swine and non-human primates) have confirmed CNS viral RNA detection, meningitis, gliosis, blood–brain barrier (BBB) injury, and enhanced systemic inflammation. In vitro systems include BBB models using human brain microvascular endothelial cells demonstrating HEV translocation, and neural cell cultures (neurons, glial cells, oligodendrocytes) supporting productive HEV replication. Together, these convergent experimental platforms provide compelling evidence that HEV can disseminate beyond the liver, cross the BBB, and infect CNS tissues across multiple species. Reproduced with modifications from [82].

Experimental models have provided compelling evidence supporting the neurotropic potential of HEV (Figure 5). Across multiple in vivo and in vitro systems, HEV has been shown to disseminate beyond the liver, cross the BBB, infect neural cells, and replicate within CNS tissues.

Among small-animal models, Mongolian gerbils experimentally infected with HEV genotype 4 developed detectable positive- and negative-strand viral RNA together with ORF2 protein expression in the brain and spinal cord, demonstrating active CNS replication [14]. Histopathological analyses revealed neuronal degeneration, Purkinje cell injury, inflammatory infiltrates, and microglial nodules, while reduced expression of ZO-1 supported BBB disruption as a mechanism facilitating CNS entry [14]. Similar findings were subsequently reproduced in rabbits infected with swine-derived HEV-4 strains, in which replicative viral RNA and ORF2 antigen were detected within neural and perivascular tissues [8]. Viral RNA and ORF2 protein have also been identified in the brains of experimentally infected mice and non-human primates, indicating that HEV neuroinvasion is not restricted to a single animal species [119].

Large-animal models further reinforced these observations. In experimentally infected pigs, both quasi-enveloped and non-enveloped genotype 3 HEV strains were detected in brain and spinal tissues and were associated with meningitis, gliosis, and perivascular inflammation [115]. Animals with CNS involvement also exhibited enhanced systemic inflammatory responses, supporting a close interaction between viral dissemination and host immune activation.

Complementary in vitro systems have confirmed that both neural and endothelial cells are permissive to HEV infection. Multiple neuronal and glial cell lines, including oligodendrocytic MO3.13 cells, support productive viral replication [13,119]. Since oligodendrocytes play a central role in myelin maintenance, their susceptibility to infection may contribute to demyelinating manifestations associated with HEV neurological disease. Human brain microvascular endothelial cells are likewise permissive to infection, and HEV exposure induces alterations in tight-junction proteins that increase BBB permeability [8].

More recently, advanced human neuron-based models have provided particularly strong evidence for HEV neurotropism. Human induced pluripotent stem cell (iPSC)-derived neurons support productive HEV infection and demonstrate preferential susceptibility of mature neurite-bearing neurons [15]. Extending these findings, human brain organoids showed sustained HEV-3 replication with persistent viral RNA release and widespread ORF2 expression across neuronal and glial populations, particularly within glutamatergic and dopaminergic neurons [120]. Together, these models provide highly relevant experimental platforms bridging conventional cell culture systems and animal models for investigating HEV-associated neurological injury.

### 5.4. Integrated Pathogenic Model

Taken together, current evidence supports an integrated pathogenic model in which HEV-associated neurological disease results from the dynamic interplay between viral neurotropism, BBB disruption, innate immune activation, adaptive autoimmunity, and intrathecal viral persistence (Figure 5). In peripheral neuropathies such as GBS and NA, immune-mediated mechanisms dominated by molecular mimicry and anti-ganglioside antibodies may predominate. Conversely, in chronic meningoencephalitis and persistent CNS infection, direct viral replication within neural tissues and compartmentalized CNS infection likely play a central pathogenic role.

Within the CNS, local activation of microglia, astrocytes, macrophages, T cells, and B cells may amplify tissue injury through a self-sustaining neuroinflammatory cascade. This integrated model helps explain the marked clinical heterogeneity of HEV-associated neurological syndromes and highlights the importance of combining virological, immunological, and neuroinflammatory perspectives when approaching diagnosis and therapeutic management.

## 6. Therapeutic Strategies for HEV-Associated Meningoencephalitis

The management of hepatitis E virus (HEV)-associated neurological manifestations, particularly meningoencephalitis, remains largely empirical, as no specific treatment guidelines are currently available. Current therapeutic strategies are mainly derived from the management of chronic HEV infection and are based on the dual objective of reducing viral replication and limiting immune-mediated neuroinflammation.

In immunocompetent patients with acute HEV infection, neurological symptoms may resolve spontaneously together with viral clearance, and treatment is primarily supportive. In contrast, in immunocompromised individuals—such as solid organ transplant recipients or patients with hematological malignancies—HEV may persist and evolve into chronic infection, increasing the risk of extrahepatic manifestations, including central nervous system (CNS) involvement. In these patients, the first therapeutic step, whenever feasible, is a reduction of immunosuppressive therapy, particularly drugs targeting T-cell function, in order to restore antiviral immune responses and facilitate viral clearance [121,122,123,124,125].

When viral persistence is established, ribavirin remains the cornerstone of antiviral therapy. Ribavirin is a nucleoside analogue that inhibits viral RNA synthesis and is administered orally, usually for at least 12 weeks, with extension guided by virological response [117,121,122,126]. Sustained virological response (SVR)—defined as persistent absence of HEV RNA in blood and stool after treatment discontinuation—is achieved in approximately 70–80% of treated patients [121,125]. However, relapse may occur in a relevant proportion of cases, even after apparent viral clearance [125]. This phenomenon has been partly attributed to viral mutations in the RNA-dependent RNA polymerase, including variants such as G1634R and Y1320H, which may alter viral fitness and response to therapy [127,128,129].

A key concept emerging from clinical and experimental studies is that of viral compartmentalization. This refers to the ability of HEV to persist in anatomically or immunologically protected sites, where antiviral drug penetration and immune surveillance are limited. Such “sanctuary sites” include the central nervous system, the gastrointestinal tract, and potentially immune cells such as PBMCs (peripheral blood mononuclear cells, including lymphocytes and monocytes) [130]. Within the CNS, the blood–brain barrier (BBB) may restrict both immune cell access and antiviral drug penetration. Consistent with this hypothesis, HEV RNA and quasispecies have been detected in cerebrospinal fluid (CSF) even after systemic clearance, and in some cases viral persistence in CSF has lasted for prolonged periods [11,12]. These findings suggest that the CNS may function as a true viral reservoir, potentially explaining delayed neurological relapses or isolated neurological disease despite systemic viral control.

Pegylated interferon-α (pegIFN-α) (a long-acting form of interferon with antiviral and immunomodulatory properties) represents an alternative therapeutic option capable of inducing viral clearance in selected cases. However, its use is limited by significant safety concerns, particularly the risk of graft rejection in transplant recipients, and therefore its application in HEV-associated neurological disease is restricted [118,124,131,132].

Among emerging antiviral strategies, sofosbuvir—a nucleotide analogue originally developed for hepatitis C virus infection—has shown inhibitory activity against HEV RNA-dependent RNA polymerase in vitro. Although synergistic effects with ribavirin have been described, clinical results remain inconsistent, and sofosbuvir monotherapy has generally failed to achieve sustained viral clearance in chronic HEV infection [133,134]. Nevertheless, isolated clinical reports have shown that combinations including sofosbuvir, ledipasvir, and ribavirin may achieve viral suppression in ribavirin-refractory cases, although the exact contribution of each drug remains unclear [135].

Beyond direct antiviral agents, increasing evidence highlights the central role of neuroinflammation in HEV-associated CNS injury. Experimental studies have demonstrated that HEV infection activates the NLRP3 inflammasome (a multiprotein intracellular complex that triggers inflammatory cytokine release) in brain endothelial cells, leading to increased production of interleukin-1β (IL-1β) and interleukin-18 (IL-18), disruption of the blood–brain barrier, and propagation of neuroinflammation [136,137,138,139,140]. These mechanisms provide a direct link between viral infection and neuronal injury through vascular and glial activation. Pharmacological inhibition of NLRP3 signaling has shown protective effects in experimental models, suggesting a potential future therapeutic target, although no clinical data are currently available in HEV-associated neurological disease [140].

Similarly, cytokine-mediated pathways contribute to CNS inflammation. Tumor necrosis factor-α (TNF-α) (a key pro-inflammatory cytokine involved in immune regulation), interleukin-6 (IL-6), and interferon-dependent signaling cascades, including the Janus kinase/signal transducer and activator of transcription (JAK/STAT) pathway (a major intracellular signaling system controlling cytokine responses), are implicated in neuroimmune regulation [141,142]. Although JAK inhibitors and biologic agents targeting these pathways are widely used in autoimmune diseases, their role in HEV-associated meningoencephalitis remains hypothetical and requires further investigation.

At the experimental level, several innovative therapeutic strategies are under investigation. These include RNA interference (RNAi) (gene-silencing technology that blocks viral RNA expression), monoclonal antibodies targeting the HEV capsid protein (ORF2), and T-cell-based immunotherapies aimed at enhancing virus-specific immune clearance [81]. Additional compounds such as heat shock protein 90 (HSP90) inhibitors, nucleotide synthesis inhibitors, and repurposed antivirals (e.g., favipiravir, gemcitabine, and azithromycin) have demonstrated anti-HEV activity in preclinical models, although none have yet reached clinical validation [143,144,145,146].

An important additional therapeutic avenue is passive immunotherapy. In selected cases of persistent or refractory HEV infection, particularly in transplant recipients, convalescent plasma therapy has been used. This approach involves administration of plasma containing neutralizing anti-HEV antibodies, thereby providing immediate passive immunity and facilitating viral clearance in patients with impaired endogenous immune responses [147]. Conceptually, this strategy is closely related to the use of immunoglobulin-based passive immunity, aiming to compensate for insufficient host antibody production and enhance viral neutralization.

Finally, prevention remains a key strategy in reducing the burden of HEV infection and its neurological complications. The recombinant vaccine Hecolin^®^ has demonstrated high efficacy in preventing HEV infection and is currently licensed in China [148,149]. Although its specific effect on neurological manifestations has not been systematically evaluated, prevention of systemic infection is expected to significantly reduce the risk of extrahepatic disease, including meningoencephalitis.

In summary, ribavirin remains the backbone of therapy for chronic or severe HEV infection, while management of HEV-associated meningoencephalitis relies mainly on supportive care and antiviral suppression. Increasing evidence of viral persistence within sanctuary sites, particularly the CNS, together with the growing recognition of neuroinflammatory mechanisms, suggests that future therapies will need to combine antiviral and immunomodulatory strategies to achieve effective disease control.

## 7. Conclusions

Hepatitis E virus has undergone a profound conceptual shift over the past two decades: from a pathogen once considered geographically restricted and purely hepatotropic, it is now recognized as a systemic agent with a broad range of extrahepatic manifestations, among which neurological complications are the most clinically significant. Meningoencephalitis, though less frequent than peripheral neuropathies such as Guillain–Barré syndrome and neuralgic amyotrophy, represents its most severe expression and poses diagnostic and therapeutic challenges only beginning to be addressed.

The pathogenesis of HEV-associated meningoencephalitis is multifactorial, combining direct viral neurotropism—with blood–brain barrier disruption, CNS invasion, and compartmentalized intrathecal replication—and a dysregulated host immune response that can sustain neurological injury independently of hepatic disease. Genetically distinct HEV quasispecies in cerebrospinal fluid, persistent viral RNA in the CNS after systemic clearance, and HEV replication in iPSC-derived neurons and brain organoids collectively support the CNS as a potential autonomous viral reservoir, with direct implications for antiviral strategy and prognosis.

Perhaps the most clinically consequential feature of HEV-associated meningoencephalitis is its frequent anicteric presentation: neurological symptoms often precede or overshadow hepatic manifestations, and liver enzyme abnormalities may be mild or overlooked, translating directly into diagnostic delays. HEV is rarely included in the routine etiological workup of unexplained encephalitis or meningitis, despite a significant proportion of such cases in Europe and other industrialized regions remaining unexplained after standard investigations. Systematic HEV serology and nucleic-acid testing—in serum and, when feasible, cerebrospinal fluid—is urgently warranted, particularly with elevated liver enzymes, immunosuppression, or relevant epidemiological exposure.

Therapeutic management remains largely empirical, and viral persistence within CNS sanctuary sites raises questions about the adequacy of current antiviral regimens. The neuroinflammatory dimension further suggests a role for combined antiviral and immunomodulatory approaches. Large prospective studies, standardized diagnostic criteria, and dedicated clinical trials remain clear research priorities.

HEV-associated meningoencephalitis is an emerging, underdiagnosed, and potentially severe condition demanding heightened clinical awareness. Every case of unexplained neurological syndrome—especially without overt jaundice—should prompt consideration of HEV as a possible etiology. Translating this awareness into routine practice remains the most urgent step toward improving outcomes for patients still too often diagnosed too late or not at all.

## Figures and Tables

**Table 1:** Clinical spectrum of hepatitis E virus-associated neurological manifestations, proposed pathogenetic mechanisms, and representative references.

Neurological Manifestation	Main Clinical Features	Proposed Pathogenetic Mechanism	Representative References
***Peripheral nervous system manifestations***			
Neuralgic amyotrophy (Parsonage–Turner syndrome)	Acute severe shoulder pain followed by brachial weakness; often bilateral	Probably immune-mediated, possible direct viral neurotropism	[100,101,102]
Guillain–Barré syndrome	Acute ascending weakness, areflexia, albuminocytologic dissociation	Immune-mediated post-infectious neuropathy	[78,103,104]
Mononeuropathies and cranial neuropathies (Bell’s palsy, facial diplegia)	Isolated peripheral nerve involvement	Immune-mediated	[105,106,107]
Myositis	Acute muscle weakness with CK elevation	Direct viral invasion and/or immune-mediated injury	[108,109]
Chronic inflammatory demyelinating polyneuropathy (CIDP)	Chronic progressive sensorimotor neuropathy	Possible immune trigger; evidence still limited	[85]
Myasthenia gravis	Autoimmune neuromuscular junction disorder	Possible triggering of autoimmunity	[84]
***Central nervous system manifestations***			
Encephalitis	Confusion, seizures, altered consciousness	Direct neuroinvasion plus immune-mediated inflammation	[90,104,110]
Meningitis/meningoencephalitis	Headache, fever, meningism with or without encephalopathy	Viral neuroinvasion; HEV RNA occasionally detected in CSF	[90,104,110]
Acute transverse myelitis	Motor, sensory and sphincter dysfunction	Direct CNS infection and immune-mediated mechanisms	[6,107]
Other CNS manifestations	Parkinsonism, recurrent myelopathy, encephalopathy	Rare; uncertain mechanisms	[87,111]

## Data Availability

No new data were created or analyzed in this study.

## Abbreviations

The following abbreviations are used in this manuscript:

AAPC	Average Annual Percentage Change
ATM	Acute Transverse Myelitis
BBB	Blood–Brain Barrier
CIDP	Chronic Inflammatory Demyelinating Polyneuropathy
CK	Creatine Kinase
CNS	Central Nervous System
CSF	Cerebrospinal Fluid
eHEV	Quasi-enveloped Hepatitis E Virus particle
ESCRT	Endosomal Sorting Complex Required for Transport
FLAIR	Fluid Attenuated Inversion Recovery
GBD	Global Burden of Disease
GBS	Guillain–Barré Syndrome
HEV	Hepatitis E Virus
HIV	Human Immunodeficiency Virus
HSP90	Heat Shock Protein 90
HVR	Hypervariable Region
IFN	Interferon
IL	Interleukin
IRES	Internal Ribosome Entry Site
IRF-3	Interferon Regulatory Factor 3
JAK/STAT	Janus Kinase/Signal Transducer and Activator of Transcription
MG	Myasthenia Gravis
MRI	Magnetic Resonance Imaging
NA	Neuralgic Amyotrophy
NCR	Non-Coding Region
NLRP3	NOD-like Receptor Family Pyrin Domain-Containing 3
ORF	Open Reading Frame
PBMC	Peripheral Blood Mononuclear Cells
PCP	Papain-like Cysteine Protease
PCR	Polymerase Chain Reaction
RdRp	RNA-dependent RNA Polymerase
RIG-I	Retinoic acid-Inducible Gene I
SDI	Socio-Demographic Index
SVR	Sustained Virological Response
TBK1	TANK-binding Kinase 1
TSG101	Tumor Susceptibility Gene 101
WHO	World Health Organization
ZO-1	Zonula Occludens-1
